# Microstructural Study on Molten Marks of Fire-Causing Copper Wires

**DOI:** 10.3390/ma8063776

**Published:** 2015-06-22

**Authors:** Kuan-Heng Liu, Yung-Hui Shih, Guo-Ju Chen, Jaw-Min Chou

**Affiliations:** Department of Materials Science and Engineering, I-Shou University; No.1, Sec. 1, Syuecheng Rd., Dashu Dist., Kaohsiung City 84001, Taiwan; E-Mails: k.heng.liu@gmail.com (K.-H.L.); yhshi@isu.edu.tw (Y.-H.S.)

**Keywords:** copper, dendrite growth, solidification microstructure, transmission electron microscopy, fire, fire investigation, fire scene, electrical short circuit, electrical arc beads

## Abstract

Although electrical fires constitute the greatest percentage of the main causes of building fires, the critical evidence used by fire investigators to identify electrical fires is not always convincing to the general public. In this study, we scrutinized the microstructures of fire-causing copper wires and simulated the external environmental conditions required for the formation of fire-causing arc beads. Our metallographic investigation revealed that the primary thermal dendrites of copper at the fire-causing arc bead grew parallel to one another, but in the opposite direction to the heat flow. We determined the relationships of the undercooling (∆*T_0_*), the growth velocity (ν), and the primary spacing (λ) of the dendrites with respect to the electrical wire’s diameter. Accordingly, fire investigators can now identify fire-causing arc beads in terms of these metallographic characteristics, thereby providing clear scientific evidence for litigant judgments of electrical fires.

## 1. Introduction

Electrical fires are among the main causes of building fires. Often, there is nothing but ash and noncombustible materials, such as metals, left at a fire scene. The critical evidence for a fire investigator to identify an electrical fire caused by a short circuit is the presence of “arc beads”, which are melted marks found at a fire scene. Such melted marks, which might be found anywhere at a fire scene, are divided into two categories: fire-melted marks (FMMs) and electric-melted marks (EMMs). The EMMs can be further divided into fire-causing arc beads (FCABs) and fire-resulting arc beads (FRABs). An FMM is a mark of an electrical wire melted as a result of the heat of the flames at a fire scene. An EMM is a melted mark formed when a huge short-circuit current surges through an electrical wire. An FCAB is a melted mark resulting from the initial short circuit that caused the fire. An FRAB is a melted mark from a short circuit that occurred on the electrical wire after the wire insulation had been burned off by the flames.

Therefore, a short circuit can cause a fire, and the fire can reciprocally enhance the damage of the short circuit. Many short-circuit arc beads might be found after a fire. It is necessary to verify whether these arc beads are FCABs or FRABs. An FMM is a large mark in the shape of a burnt melted drop; an FCAB is a much glossier and finer bead; and an FRAB is a non-glossy rough bead [1]. The differences between an FMM and an EMM can be distinguished easily by comparing the melted ranges and shapes, but distinguishing between an FCAB and an FRAB can be difficult. Fire investigators in fire departments typically attempt to identify melted marks through visual inspection with reference to the literature [1], even though the resolution of the human eyes is only approximately 62.5 μm and macroscopic comparison can be quite subjective. Identification based on visual observation of the size and color of an arc bead remains controversial.

Using scanning electron microscopy (SEM), Gray *et al.* [2] observed numerous square and rectangular pockmarks on FCABs. Erlandsson and Strand [3] found copious voids in the cross sections of arc beads. A study by the Tokyo Fire Department revealed that even arc beads formed in the air at 1000 °C contained voids, albeit smaller and more likely to be near the surface of the FCAB. On the other hand, large voids have been observed deeper inside FRABs [4]. Lee *et al.* [5] found that the spacing between the dendrite arms reflected the ambient temperature where the beads solidified; thus, the dendrite arm spacings of FCABs were smaller than those of FRABs.

The identification of arc beads remains controversial. Until now, there has been no scientific evidence regarding FCABs available for investigations [6,7]. Therefore, concrete scientific evidence still cannot be provided in judicial courts for cases related to electrical fires [8]. Thus, the evidential power of the investigation affects the judicial prosecution, litigation, and judgment of fire cases. Because the public reliance on FCAB verification is also uncertain, urgent issues must be addressed by fire agencies [9].

In this study, we simulated the external environmental conditions required for the formation of FCABs; herein, we discuss their effects on the metallography of electrical wires based on materials science especially, solidification theory. We investigated the relationships among the primary spacing of dendrites (λ), the growth velocity (ν) and the temperature difference due to heat flow (∆*Tt*) of fire-causing samples at various wire diameters (*D*).

The primary spacing (λ) of thermal dendrites after the solidification process reflects the cooling rate of the melted metal. We determined the relationship between the wire diameter and the primary spacing of thermal dendrites to explore the effects of the steady-state growth-tip radius (*R_S_*), extremum radius (*Re*), growth velocity (ν), and the temperature difference due to heat flow (∆*Tt*) from the primary trunks of various thermal dendrites.

In this study, we used X-ray diffraction (XRD), focused ion beam (FIB), SEM, energy dispersive spectroscopy (EDS), and transmission electron microscopy (TEM), as well as corresponding mathematical models, to examine the microstructural constituents of FCABs. We believe that our results will improve the scientific basis for identifying FCABs. This microstructural study of FRABs is ongoing; we will provide comparisons of FCABs and FRABs in future studies.

## 2. Experiment

### 2.1. Experimental Materials

Because the short-circuiting of multiple-conductor wires involves some complicated factors, single-conductor wires were used in this study to simplify the calculations. The materials used for the short-circuit experiments were copper electric wires (99.99% purity) having diameters of 0.1, 0.2, 0.4, 0.6, 0.8, and 1.2 mm, respectively. Two copper electric wires (length: 10 cm) were peeled off from each group (two wires per group) to form the loops in the short-circuit experiments. The wire diameter of the branch circuit used for a general house is approximately 2.0 mm, the short-circuit current of the 1.2 mm wire in diameter was as large as 750 amp in this experiment. Thus, for safety considerations, the 1.2 mm wire was set as the largest diameter in this study.

### 2.2. FCAB Sample

**Figure 1:**
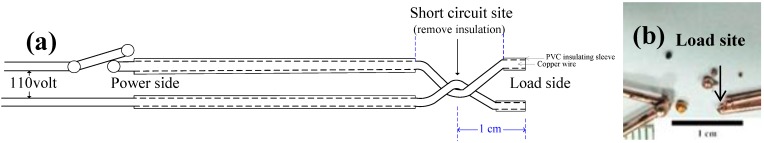
Short-circuit experiment: (**a**) schematic diagram; (**b**) fire-causing arc bead (FCAB) samples.

Short-circuiting of the electrical wires under ambient atmosphere generated FCABs. The blowout and solidification of the electrical wires occurred instantaneously in response to the short-circuiting of the loaded wire, even without an ignition of fire. Therefore, in this experiment, the short site was preset 1 cm from one end (load side). The insulating PVC layer of the preset short site was peeled off and two wires were twisted to make contact. The contacting copper wires were placed in ambient atmosphere, and the other end (power side) was connected to an AC power supply (110 V, 60 Hz), as displayed in Figure 1. When the power supply was turned on, the two copper wires sparked instantly due to the short circuit, until the short-circuit current blew out the two conductors. The arc bead samples were obtained from the blown part of the wire.

### 2.3. Analytical Methods

No short-circuit current passed through the load-side wire as the short circuit occurred, at room temperature; thus, strong heat extraction occurred on the load-side wire, producing a large undercooling, which may have caused a phase transformation in the melt of the molten mark. Therefore, the short-circuit arc bead samples on the load side were soaked in acetone, and then cleaned using an ultrasonic oscillator. The cleaned arc bead samples were then placed in a moisture-proof box at room temperature for natural drying.

The morphologies and compositions of the dried samples were investigated using field-emission SEM (Hitachi S-4700, Tokyo, Japan); XRD analysis of the samples was performed using an XRD diffractometer (PANalytical, Almelo, The Netherlands).

The microstructures of the samples were observed using focused ion beam SEM (Seiko SMI3050SE dual-beam FIB-SEM hybrid system, Oyama, Japan). The sampling sites were cut, using a gallium ion beam, to prepare the TEM specimens. The chemical compositions of the specimens were analyzed by SEM with attached EDS (METEK, Gloucestershire, UK, 5 ≤ *Z* ≤ 92).

The electronic diffraction patterns, including bright- and dark-field images, of the specimens, were recorded using TEM (FEI Tecnai G^2^ 20 S-Twin, Hillsboro, OR, USA). The distances (*L*) between the diffraction spots and transmitted electron beam in the diffraction patterns were measured to determine their crystallographic relationship. Because the reciprocal of a reciprocal lattice-vector length is the interplanar spacing (*d*) of the crystal plane (hkl), the inter-planar spacing corresponding to the diffraction spot was calculated according to the value of L. Equations (1)–(4) were then derived from the equation of Ld=const. [10] as well as the inner vector products:

(1) $$L_{1}:L_{2}:L_{3}:L_{4}=\sqrt{h_{1}^{2}+k_{1}^{2}+l_{1}^{2}}:\sqrt{h_{2}^{2}+k_{2}^{2}+l_{2}^{2}}:\sqrt{h_{3}^{2}+k_{3}^{2}+l_{3}^{2}}:\;\sqrt{h_{4}^{2}+k_{4}^{2}+l_{4}^{2}}$$

(2) $$\left[h_{1}k_{1}l_{1}\right]\cdot \left[h_{2}k_{2}l_{2}\right]=\sqrt{h_{1}^{2}+k_{1}^{2}+l_{1}^{2}}\cdot \sqrt{h_{2}^{2}+k_{2}^{2}+l_{2}^{2}}\cdot \cos\theta_{21}$$

(3) $$\left[h_{1}k_{1}l_{1}\right]\cdot \left[h_{3}k_{3}l_{3}\right]=\sqrt{h_{1}^{2}+k_{1}^{2}+l_{1}^{2}}\cdot \sqrt{h_{3}^{2}+k_{3}^{2}+l_{3}^{2}}\cdot \cos\theta_{31}$$

(4) $$\left[h_{1}k_{1}l_{1}\right]\cdot \left[h_{4}k_{4}l_{4}\right]=\sqrt{h_{1}^{2}+k_{1}^{2}+l_{1}^{2}}\cdot \sqrt{h_{4}^{2}+k_{4}^{2}+l_{4}^{2}}\cdot \cos\theta_{41}$$

Various plane indices (hkl) were obtained Equations (1)–(4); the structures and phase identifications of the constituents were then identified after comparison with the inter-planar spacing data from the Joint Committee on Powder Diffraction Standards (JCPDS) cards [04-0836].

The arc bead samples were mechanically ground and soaked for 60 s in an etchant solution [25% ammonium hydroxide (5 mL), 30% hydrogen peroxide (2 mL), pure water (5 mL)]. The samples were removed for cleaning and drying and then a metallurgical microscope was used to observe the morphologies of the cross sections of the arc bead samples.

## 3. Results

When copper electrical wires were short-circuited and blown out, the melted copper liquid formed arc beads and marks at the blown part of the wire under the effect of surface tension [11].

**Figure 2:**
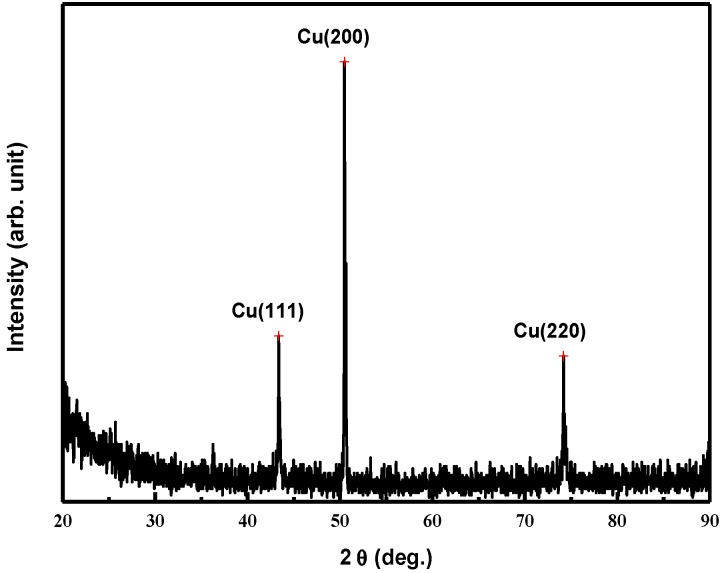
X-ray diffraction (XRD) analysis of the arc bead samples.

The XRD patterns of the arc bead samples (Figure 2) revealed peaks at values of 2θ of 43.33°, 50.47°, and 74.20° representing the (111), (200), and (220) planes respectively, of pure copper. Because the growth rates of high-index planes with high surface energy were quicker than those of low-index planes in the solidified copper, the low-index planes of copper were left in the final solidification. The peak at a value of 2θ of 50.47° was the strongest, suggesting that the (200) plane was the preferred crystallographic plane.

**Figure 3:**
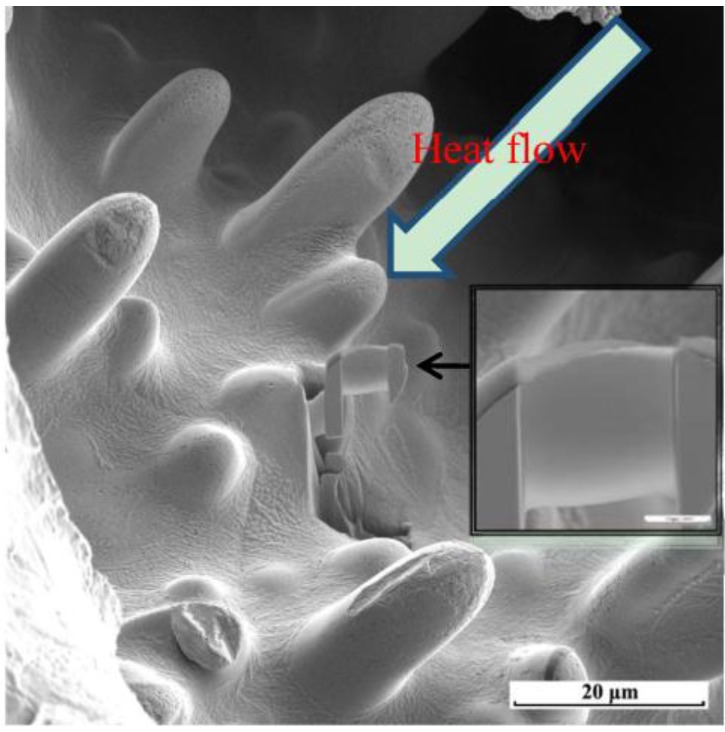
Microstructures of arc bead samples observed through focused ion beam SEM (FIB-SEM) and the sampling of a transmission electron microscopy (TEM) specimen.

**Figure 4:**
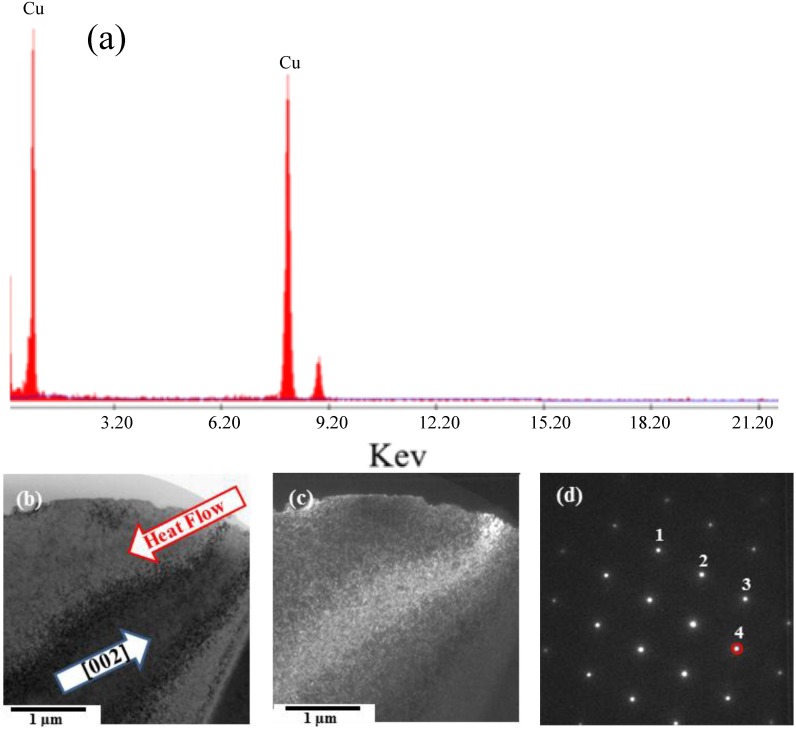
TEM analysis of primary crystallites: (**a**) energy dispersive spectroscopy (EDS) spectrum; (**b**) TEM bright-field image; (**c**) dark-field image and (**d**) diffraction pattern along the [0$\bar{1}$1] direction.

The surface morphologies of the arc bead samples exhibited the single-phase growth of primary dendritic crystals. Figure 3 reveals that the primary dendrites possessed a non-faceted growth surface morphology, with outward growth derived from the unfused wire [12]. We used a FIB for ion-bombardment cutting of the primary dendrite tips to prepare TEM specimens. The main element in the primary crystal specimens was copper (Figure 4a). We employed electron diffraction through TEM for structural analysis of the primary crystal specimens (Figures 4b–d), measuring the values of *L* and θ from the diffraction pattern and calculating the corresponding plane indices (*hkl*) from Equations (1)–(4). Matching the data from JCPDS cards to the interplanar spacing confirmed that the primary crystal was a copper phase with a face-centered cubic crystal structure and a lattice constant of 0.3615 nm (Table 1).

**Table 1:** Interplanar spacings of dendrite specimens calculated from the electron diffraction pattern.

Spot	Calculated Interplanar Distance (Å)	Standard Interplanar Distance (Å)	Crystalline Plane (*hkl*)
1	1.2728	1.2780	$\left(0\bar{2}\bar{2}\right)$
2	2.0859	2.0880	$\left(1\bar{1}\bar{1}\right)$
3	1.8034	1.8080	(200)
4	2.0864	2.0880	(111)

**Figure 5:**
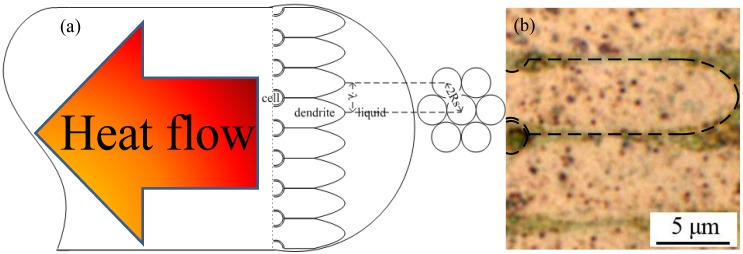
Growth of primary trunk of thermal dendrites in a melted bead (anti-parallel to the heat flow direction); (**a**) schematic diagram of the thermal dendrites; (**b**) metallographic photo of thermal dendrites in arc bead of 0.6mm wire.

Although plate-like primary crystallites are often observed during solid-state precipitation, the primary crystallites in our present experiment grew into rod-like shapes. The conducting wire was a good thermal extracting material. Heat from the melted copper liquid was extracted into the super-cooled melt through a conduction effect; the solid-liquid interface generated a temperature inversion [13,14]. As the latent heat was released from the solid-liquid interface, the temperature at the interface became hotter than that on the liquid side. Therefore, an unstable interface appeared because the interface front produced a negative temperature gradient that led to part of the heat flowing toward the super-cooled liquid [15]. When the crystallization of copper occurred toward the super-cooled liquid region, the primary dendrites crystallized rapidly in rod-like form, while the secondary branch cell body grew in the <001> preferred direction, out of the surface where the primary trunk contacted the super-cooled liquid. The latent heat released from the dendrite growth of the secondary branch increased the temperature of the liquid nearby obstructing the formation of other protruding interfaces [16]. As a result, the secondary branch dendrites competed with, and restricted, one another in terms of their growth. The direction anti-parallel to the heat flow became the dominant growth direction. Therefore, the primary trunks of the thermal dendrites grew in a parallel manner to form the columnar crystalline structure displayed in Figure 5. The formation of thermal dendrites is the unique fingerprint of FCABs in the load-side wire.

**Figure 6:**
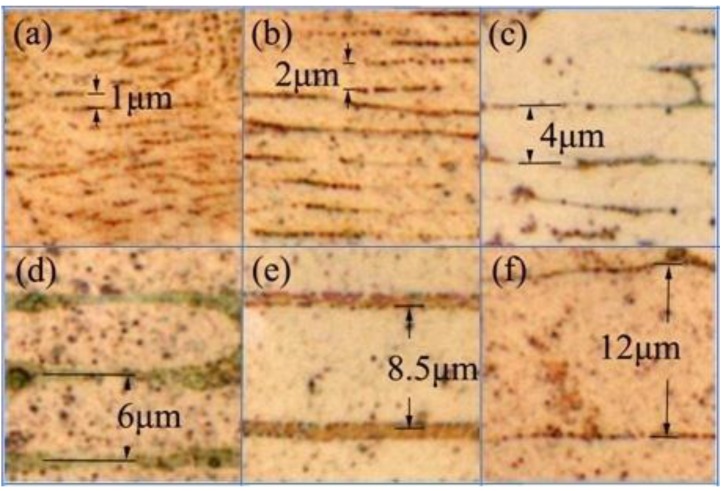
Cross-sectional metallographic analyses of arc beads of copper wire with diameters of (**a**) 0.1, (**b**) 0.2, (**c**) 0.4, (**d**) 0.6, (**e**) 0.8, and (**f**) 1.2 mm.

We repeated the short-circuit experiments 10 times for the wires of each diameter. The samples were obtained from two ends of the load-side wire at the short-circuit site. We used metallography to measure the values of λ of the FCABs at five sites for each sample as shown in Figure 6. The sampling number, *N*, was more than 100 for the FCABs at each wire diameter. Table 2 lists the average value of λ of the FCABs at each diameter, λ_ɑve_, and the standard deviations, $\text{SD}=\sqrt{1/N\;\sum_{i=1}^{N}{\left(\lambda_{i}-\lambda_{ave}\right)}^{2}}$, are calculated.

**Table 2:** Average primary spacings (λ_ɑve_), growth velocities (ν_s_), and the temperature differences (∆*Tt_S_*) of thermal dendrites of copper arc beads obtained from wires of various diameters.

Parameter	Correspondind Datum					
Diameter of Wire (mm)	0.1	0.2	0.4	0.6	0.8	1.2
λ_ɑve_ (μm)	0.83 ± 0.17	1.85 ± 0.36	3.59 ± 0.70	5.92 ± 0.95	8.43 ± 1.41	11.83 ± 1.61
ν_s_ (μm/s)	3.58 × 10^6^	8.95 × 10^5^	2.24 × 10^5^	9.94 × 10^4^	5.59 × 10^4^	2.48 × 10^4^
∆*Tt_S_* (K)	10.09	5.04	2.52	1.68	1.26	0.85

## 4. Discussion

Although the kinetics of atomic addition plays a key role in some materials, we assumed that the diffusion of atoms from the liquid phase to the crystalline phase was rapid so that the kinetics model could be neglected [17,18,19,20,21,22] because the dendrite morphologies clearly exhibited “non-faceted” growth.

Because heat diffusivity is much faster than mass diffusivity [23], the solid-liquid interface of a pure metal will always be unstable if the temperature gradient is negative. Perturbations will grow if the temperature gradient is negative at the interface, resulting from the heat flux due to the temperature gradient (*G* = d*T*/d*z*) [24]. The total undercooling (∆*T*_0_) of the melt is equal to the difference between *T_m_* (the melting point) and *T_l_* (the liquid temperature).

**Figure 7:**
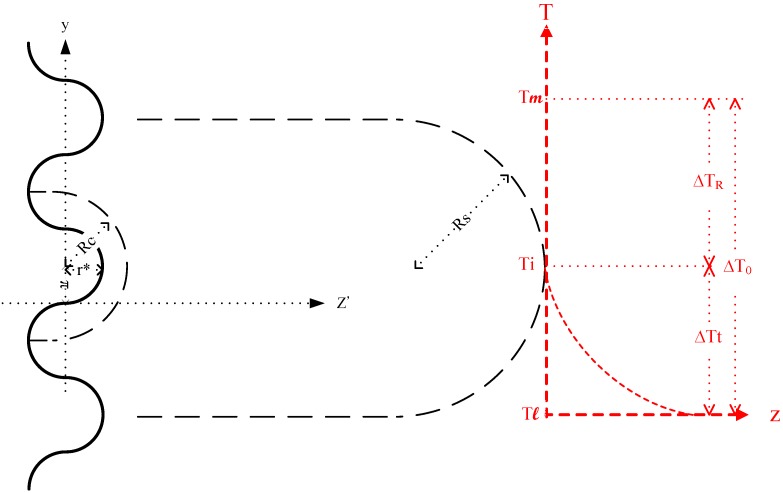
Schematic representation of dendrite growth initiated by perturbation at the solid–liquid interface, and the corresponding temperature curve in front of the dendrite tip.

The perturbations have an infinitesimal critical amplitude r*, a quarter wave length, which does not affect the diffusion fields. The perturbed interface can be described by a simple sine function (Figure 7):

z′=r*sin(ωy)

where ω = π/2r* is the wave number.

The interface temperature *T_i_* can be deduced from the assumption of local equilibrium:

$$T_{i}=T_{m}-\Delta T_{R}$$

here *T_i_* equals the difference between the melting point (*T*_m_) and the curvature undercooling ∆*T*_R_ = Γ*K*, where the Gibbs–Thomson coefficient (Γ*)* is equal to γ∆𝒽_f_/*T*_m_, and the curvature *K* is $r^{*}{\text{ω}}^{2}\sin(\text{ω}y)$; γ is the interface energy and ∆𝒽_f_ is the latent heat.

The temperature difference between the tips (at point *t*) and depressions (at point *d*) of the interface is given by:

$$\begin{array}{l} T_{t}-T_{d}=-\Gamma \left(K_{t}-K_{d}\right)\\ ∵\;G=\frac{T_{t}-T_{d}}{2r^{*}}\\ ∴\;r^{*}=\frac{\pi }{2}\sqrt{\frac{\Gamma }{\left(-G\right)}} \end{array}$$

When a protrusion appears on the solid (Figure 7), the negative temperature gradient in the liquid becomes even more negative. The advancing perturbations then continuously evolve into the cell, with the cell radius *Rc* having twice the value of r* at ∆*t_0_*, $Rc=\text{π}\sqrt{\frac{\Gamma }{\left(-G_{c}\right)}}$.

The tip growth at the cell is isothermal; the thermal diffusion equation in cylindrical coordinates at the solid-liquid interface of the growing tip can be written [25,26]:

$$\frac{\partial T}{\partial 𝓉}=\text{a}\left(\frac{\partial^{2}T}{\partial R^{2}}+\frac{2}{R}\frac{\partial T}{\partial R}\right)$$

$$G_{c}={\left(\frac{\text{d}T}{\text{d}R}\right)}_{R_{c}}=\frac{-\Delta Tt}{R_{c}}$$

*a*: thermal diffusivity; ∆*Tt:* the temperature difference due to heat flow.

The heat moving away from the interface through the liquid must balance the sum of the heat from the solidification and the latent heat generated at the interface:

$$\nu △{𝒽}_{\text{f}}=\text{a}𝒸\left(\frac{△Tt}{R_{C}}\right)$$

ν*:* growth velocity; *c*: volumetric specific heat.

The cell grows continuously into dendrite, due to the temperature difference ∆*Tt* at the tips of the solid–liquid interface. From the Gibbs–Thomson effect, the equilibrium across the dendritic tip radius occurs at a curvature undercooling (∆*T*_R_) below the melting point *T*_m_ is given by $\Delta T_{R}=\frac{2\gamma T_{𝓂}}{R\Delta {𝒽}_{\text{f}}}$.

$$∴\frac{\Delta T_{R}}{\Delta T_{0}}=\frac{Rc}{R}$$

The total undercooling of melt $\Delta T_{0}$ equals the sum of the undercooling due to curvature (∆*T_R_*) and the temperature difference from the heat flow (∆*Tt*):

$$\begin{array}{l} ∴\Delta T_{t}=\Delta T_{0}-\Delta T_{R}\\ \nu =\frac{ac△T_{0}}{△{𝒽}_{f}R}\left(1-\frac{\Delta T_{R}}{\Delta T_{0}}\right) \end{array}$$

The growth of a thermal dendrite at marginal stability is given by $\frac{\text{d}\nu }{\text{d}R}=0$

$$∴R_{S}=2Rc$$

*R_s_:* dendrite radius at marginal stability.

The primary trunk tip radius of thermal dendrite growth at marginal stability is obtained [27,28,29,30,31]:

(5) $$∴\;R_{S}=2\pi \sqrt{\frac{\Gamma }{\left(-G_{S}\right)}}$$

(6) $$Gs={\left(\frac{dT}{dR}\right)}_{R_{s}}=\frac{-△Tt_{s}}{R_{s}}$$

*Gs*: temperature gradient of dendritic tip at marginal stability; ∆*Tt_s_*: temperature difference of dendritic tip at marginal stability due to heat flow.

**Figure 8:**
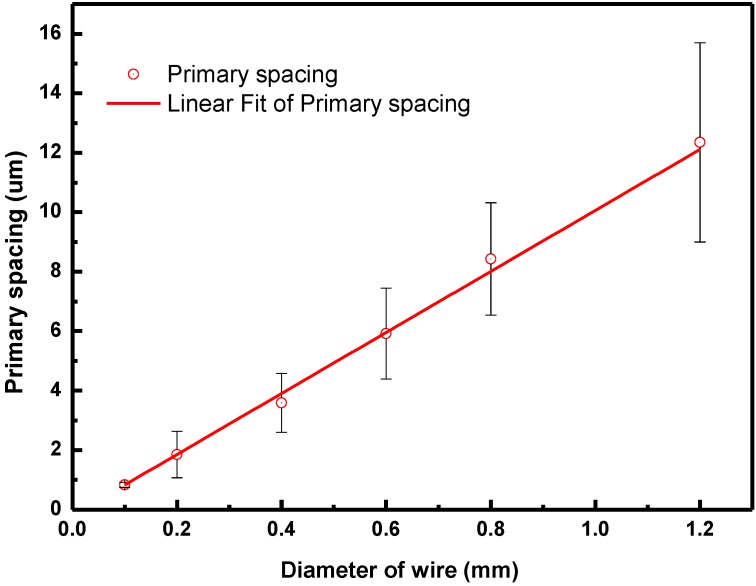
Relationship between the primary spacings of thermal dendrites and the diameter of the wire.

Our experiment revealed the relationship between the wire diameter and the primary spacing of the thermal dendrite of the short-circuit arc bead samples, as illustrated in Figure 8. From the microstructure, the *D*–λ relationship between the wire diameter and the primary spacing of the thermal dendrite is obtained as follows:

(7) $$\lambda ≒10^{-2}D$$

Meanwhile, the stability limit of the thermal dendrite tip radius *R_s_* is proportional to λ. The proportionality constant depends on the geometrical arrangement of the dendrites grown in steady state. From the hexagonal arrangement in Figure 5, it is likely that the last liquid solidifies at the gravity center of the equilateral triangle that is formed by the three densely packed dendrites [31]. This assumption leads to the value of

(8) $$\lambda =\sqrt{3}R_{S}$$

A cylinder having a hemispherical tip (Figure 7) growing along its axis is the simplest approximation that can be adapted to the growth of a dendrite tip [32,33]. The cross-section of the cylinder, $A=\text{π}R_{S}^{2}$, determines the volume that grows in a time 𝒹𝓉 and is responsible for the rejection of thermal heat. The surface area of the hemispherical cap, $A_{S}=2\text{π}R_{S}^{2}$, determines the amount of radial thermal diffusion. Thus, the flux due to thermal rejection *J*_1_ equals the thermal diffusion in the liquid ahead of tip (*J*_2_) which can be written by:

$$A\nu_{s}△{𝒽}_{f}=A_{S}a𝒸\left(\frac{△Tt_{s}}{R_{s}}\right)$$

ν*_s_*: growth velocity of thermal dendrite at marginal stability.

Under steady-state conditions, the heat flux balance must lead to the thermal Péclet numbers (*P_t_*) which are defined by the dimensionless ratio of the radius *R* to the thermal-diffusion distance 2*a*/ν at thermal diffusion-limited growth [34,35,36,37]:

(9) $$P_{t_{s}}=\frac{R_{s}\nu_{s}}{2\text{a}}$$

Because the stability limit of the dendrite tip lies at a larger tip radius than that of the extremum, the capillarity effect can be neglected [38,39].

At marginal stability, the thermal supersaturation Ω can be defined as the ratio of the thermal undercooling to the unit undercooling (θ_*t*_ = ∆𝒽*_f_/c*) The relationship defining the thermal diffusion at the tip for the marginally stable growth of a thermal dendrite is given by $\text{Ω}=P_{t}$. Thus, the temperature difference due to heat flow can be written as ∆*Tt* = *P_t_*θ*_t_*; the temperature difference due to the heat flow of dendrite growth at stability is obtained by

(10) $$\Delta Tt_{s}=P_{t_{s}}{\text{θ}}_{t}$$

Combining Equations (6), (9), and (10) into Equation (5), the growth velocity and the radius of thermal dendrite growth at marginal stability are obtained respectively by

(11) $$\nu_{s}=\frac{8{\text{π}}^{2}\text{aΓ}}{\theta_{t}R_{S}^{2}}$$

(12) $$R_{s}=2\pi \sqrt{\frac{2\text{aΓ}}{\nu_{s}\theta_{t}}}$$

Table 2 lists the growth velocities (ν*_s_*) of the thermal dendrites in the arc beads. The growth velocities of the thermal dendrites of the melted beads having diameters ranging from 0.1 to 1.2 mm are inversely proportional to the square of the wire diameter. That is, as the diameter of the wire increases, the growth velocity of the thermal dendrite decreases.

By combining Equations (9) and (12) into Equation (10), ∆*Tt_s_* is obtained:

(13) $$\Delta Tt_{s}=\sqrt{\frac{2\pi^{2}\Gamma \theta_{t}\nu_{s}}{\text{a}}}$$

As listed in Table 2, the values of ∆*Tt_s_* due to the thermal dendrite growth of the melted beads having diameters from 0.1 to 1.2 mm are inversely proportional to the wire diameters. Thus, when the diameter of the wire increases, the value of ∆*Tt_s_* also decreases.

The extremum growth of a thermal dendrite is influenced by diffusion, capillarity effects, and the degree of thermal supersaturation (Ω). This thermal supersaturation is defined by the equation

*Ω_e_ = Pt_e_ + 2Γ/R_e_θ_t_*

The temperature difference ∆*Tt_e_* due to the heat flow of extremum growth is equal to Ω*_e_*θ*t*. The extremum of growth velocity (ν*_e_*) is used to define the tip radius (*R_e_*). The maximum velocity in an isothermal environment corresponds to a minimum in the total undercooling (∆*T_0_*) for constant-velocity growth. Therefore, minimizing ∆*T_0_* will give the extremum radius (*R_e_*) and the temperature difference due to heat flow (∆*Tt_e_*):

$$\frac{\text{dΔ}T_{0}}{\text{d}R}=0$$

(14) $$R_{e}=2\sqrt{\frac{\Gamma a}{\nu_{e}\theta_{t}}}$$

(15) $$\text{Δ}Tt_{e}=2\sqrt{\frac{\Gamma \nu \theta_{t}}{\text{a}}}$$

**Figure 9:**
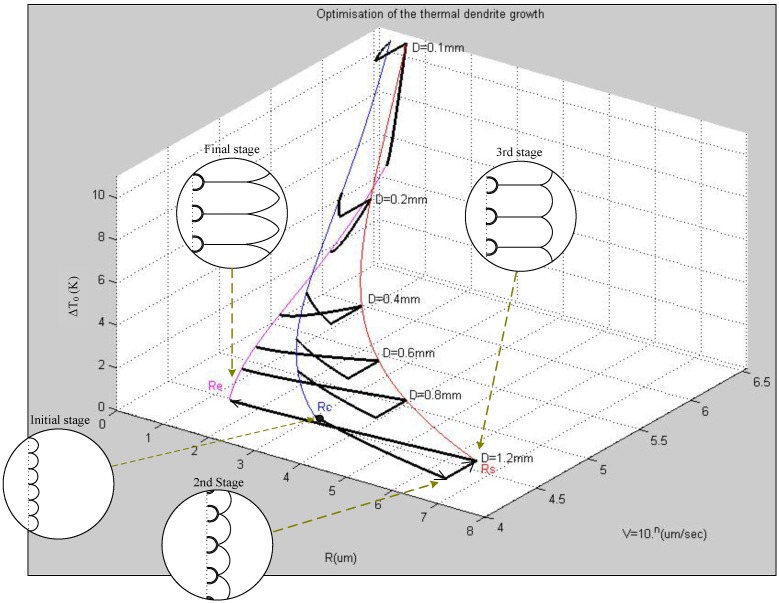
Relationships among the values of Δ*T*_0_, *R*, and ν, of the thermal dendrites obtained from various wire diameters (*D*) during different growth stages.

Figure 9 presents the overall relationships between the wire diameter (*D*), the total undercooling (∆*T*_0_), the tip radius (*R_s_*, *R_e_*), and the growth velocity (*ν*) of thermal dendrites at various growth stages.

## 5. Conclusions

From short-circuit experiments performed at ambient atmosphere, we studied FCABs located 1 cm away from the load end of wires having diameters from 0.1 to 1.2 mm. The total undercooling (∆*T_0_*) of the melted bead grown out of the thermal dendrite was inversely proportional to the wire diameter. The thermal dendrite growth velocity (ν) was inversely proportional to the square of the wire diameter. The primary spacing of the dendrite (λ) is proportional to the wire diameter. Therefore, the total undercooling (∆*T_0_*) of the melt of an arc bead, as well as the growth velocity (ν) of thermal dendrite increased upon decreasing the diameter of the wire. Thus, decreasing the diameter of the wire increased the cooling rate of the arc-bead melt. Our aim for this study was to obtain evidence for FCABs from various perspectives of materials science and to derive a corresponding mathematical model. Indeed, we successfully deduced several interacting physical properties: the primary spacing of thermal dendrite (λ), the growth velocity (ν), and the total undercooling (∆*T_0_*) in the arc beads, all with respect to the diameter of the copper wire. Accordingly, instead of merely applying visual identification or empirical rules, FCABs can be verified scientifically, thereby effectively enhancing the evidential power in judicial fire investigations.
